# Novel scintillating material 2-(4-styrylphenyl)benzoxazole for the fully digital and MRI compatible J-PET tomograph based on plastic scintillators

**DOI:** 10.1371/journal.pone.0186728

**Published:** 2017-11-27

**Authors:** Anna Wieczorek, Kamil Dulski, Szymon Niedźwiecki, Dominika Alfs, Piotr Białas, Catalina Curceanu, Eryk Czerwiński, Andrzej Danel, Aleksander Gajos, Bartosz Głowacz, Marek Gorgol, Beatrix Hiesmayr, Bożena Jasińska, Krzysztof Kacprzak, Daria Kamińska, Łukasz Kapłon, Andrzej Kochanowski, Grzegorz Korcyl, Paweł Kowalski, Tomasz Kozik, Wojciech Krzemień, Ewelina Kubicz, Mateusz Kucharek, Muhsin Mohammed, Monika Pawlik-Niedźwiecka, Marek Pałka, Lech Raczyński, Zbigniew Rudy, Oleksandr Rundel, Neha G. Sharma, Michał Silarski, Tomasz Uchacz, Wojciech Wiślicki, Bożena Zgardzińska, Marcin Zieliński, Paweł Moskal

**Affiliations:** 1 Faculty of Physics, Astronomy and Applied Computer Science, Jagiellonian University, Kraków, Poland; 2 Institute of Metallurgy and Materials Science of Polish Academy of Sciences, Kraków, Poland; 3 INFN, Laboratori Nazionali di Frascati, Frascati, Italy; 4 Institute of Chemistry, University of Agriculture, Kraków, Poland; 5 Institute of Physics, Maria Curie-Sklodowska University, Lublin, Poland; 6 Faculty of Physics, University of Vienna, Vienna, Austria; 7 Faculty of Chemistry, Jagiellonian University, Kraków, Poland; 8 Departament of Complex System, National Centre for Nuclear Research, Otwock-Świerk, Poland; 9 High Energy Physics Division, National Centre for Nuclear Research, Otwock-Świerk, Poland; 10 Department of Physics, College of Education for Pure Sciences, University of Mosul, Mosul, Iraq; University of Chicago, UNITED STATES

## Abstract

A novel plastic scintillator is developed for the application in the digital positron emission tomography (PET). The novelty of the concept lies in application of the 2-(4-styrylphenyl)benzoxazole as a wavelength shifter. The substance has not been used as scintillator dopant before. A dopant shifts the scintillation spectrum towards longer wavelengths making it more suitable for applications in scintillators of long strips geometry and light detection with digital silicon photomultipliers. These features open perspectives for the construction of the cost-effective and MRI-compatible PET scanner with the large field of view. In this article we present the synthesis method and characterize performance of the elaborated scintillator by determining its light emission spectrum, light emission efficiency, rising and decay time of the scintillation pulses and resulting timing resolution when applied in the positron emission tomography. The optimal concentration of the novel wavelength shifter was established by maximizing the light output and it was found to be 0.05 ‰ for cuboidal scintillator with dimensions of 14 mm x 14 mm x 20 mm.

## Introduction

Plastic scintillators are commonly used as radiation detectors in particle and nuclear physics experiments. Low cost of manufacturing, possibility of forming in different shapes and opportunity to acquire relatively high light output renders plastic scintillators suitable for cost-effective construction of large size detectors [1]. Recently novel cost-effective method of the whole-body positron emission tomography was developed by the Jagiellonian Positron Emission Tomography Collaboration [2–5]. The main novelty of the method is the application of long strips of low-cost plastic scintillators instead of small crystals as detectors of annihilation gamma quanta [6–9]. To date, all commercial PET scanners use inorganic crystals as detectors of annihilation gamma quanta. Plastic scintillators were not applied because of their low probability of photoelectric effect and therefore lower detection probability compared to inorganic crystals [10, 11]. This disadvantage is compensated in the J-PET scanner by the large geometrical acceptance, superior timing properties, and utterly different method of determination of gamma quanta interaction place that is based on the time when scintillation pulse has been registered with photodetectors.

**Fig 1:**
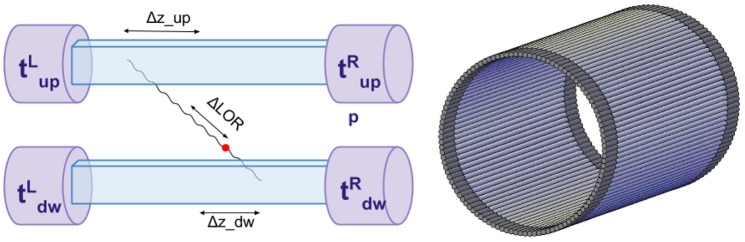
J-PET detection modules: A pair and a single layer. (A) Left panel: scheme of the pair of J-PET detection modules. Such pair constitutes a simplest detection unit capable of registration both collinearly propagating quanta resulting from electron—positron annihilation. Times of light signals arrivals to photomultipliers are denoted as: $t_{up}^{L}$—to left upper photomultiplier, $t_{up}^{R}$—to right upper photomultiplier, $t_{dw}^{L}$—to left lower photomultiplier, $t_{dw}^{R}$—to right lower photomultiplier. Red dot indicates a place of annihilation, Δ*z* denotes the distance between the point of interaction in scintillator and the center of the strip, Δ*LOR* denotes the position of annihilation along the line-of-response between two strips with respect to the centre of LOR. Both Δ*z* and Δ*LOR* are determined based on the measured times $t_{up}^{R}$, $t_{up}^{L}$, $t_{dw}^{L}$, $t_{dw}^{R}$. (B) Right panel: schematic visualization of an example of a single detection layer of the J-PET detector. Each scintillator strip is aligned axially and read out at two ends by photomultipliers.

In the recent publications [2–4], it was shown that the PET based on plastic scintillators may even outperform the classical crystal PET also as regards sensitivity. TOF resolution for the detector of 30 cm length is better by about a factor of two with respect to the current TOF-PET tomographs characterized by typical field of views of about 16 cm. In the novel approach, developed by J-PET collaboration, timing of the photodetector signals is used instead of their amplitude information. To improve the sampling rates of the fast signals in the voltage domain, solely digital, front-end signal processing electronics was developed [12–14]. Compressing sensing [15, 16] and method based on the library of synchronized model signals [3, 17] are used for the hit-time and hit-position reconstruction. A single module of the J-PET detector consists of a scintillator strip read out at two ends by photomultipliers (left side of Fig 1) [2]. The gamma quantum hit position is determined from the difference of times of arrival of the light signals to the ends of the scintillator. The annihilation point (indicated as red dot) along the LOR (Line of Response) is calculated as $\Delta LOR=0.5(t_{up}^{hit}-t_{down}^{hit})c$, where c denotes a speed of light, $t_{up}^{hit}$ and $t_{down}^{hit}$ denote hit-times of gamma quanta in the upper and lower strip as indicated in the left side of Fig 1. Right side of Fig 1 illustrates one of the possible arrangements of scintillator strips in the J-PET tomograph. Such construction permits to enlarge the length of the field-of-view of the tomograph by increasing the length of the scintillator strips without rising of the cost of photomultipliers and electronics.

It permits also to combine PET and Magnetic Resonance Imaging (MRI) in a unique way [18, 19], complementary to presently applied PET/MRI solutions [20, 21] such that both metabolic and morphological images can be registered at the same place and at the same time. This is possible by inserting a non-magnetic plastic PET barrel inside a MRI tomograph. Such solution requires however usage of long strips of scintillators and silicon photomultipliers (SiPM) whose operation is not disturbed by the magnetic field. Application of the matrix readout of SiPM instead of vacuum tube photomultipliers would be also an advantage in terms of improvement of the time and spatial resolution. In the reference [4] it was shown that recording timestamps of several photons, at two ends of the scintillator strip, by means of SiPMs matrix may improve time and spatial resolution by more than factor of 1.5. The time resolution decreases however with the increase of the length of the scintillator strip due to the light attenuation. Bulk light attenuation length of plastic scintillators e.g. BC-420 (Saint Gobain) is claimed by the producer to be about 100 cm [22], however in practice the effective attenuation of light signals in strips with finite cross section is much lower. This is due to the fact that photons on a way from the emission point to the end of the scintillators undergoes many internal reflections and their optical length is much longer than the length measured along the scintillator. For example, for strips of BC-420 scintillator with cross section of 19 mm x 5 mm the attenuation length amounts to about 37 cm only [23].

In this article we describe results of investigations aiming at the development of a new scintillating material which would be characterized by the timing properties similar to the presently known plastic scintillators but whose photon emission spectrum would be extended towards longer wavelengths, thus providing lower light attenuation and more efficient matching to the quantum efficiency of the SiPM converters.

In the following sections we first describe method of manufacturing and composition of a novel material, further on referred to as J-PET scintillator. Subsequently optical properties and molecular weight of the J-PET scintillators will be discussed in view of its application for PET/MRI scanner. Next light output and characteristics of scintillation signals will be determined and compared to the properties of commercially available scintillators.

## Materials and methods

### Scintillator composition and the polymerization method

Plastic scintillators usually consist of three components: polymeric base, primary and secondary additives. The scintillation mechanism lies in energy transfer between those components. Firstly, polymer molecules are excited by the radiation e.g. gamma quanta. Next, this excitation energy is transferred to the primary additive via Förster mechanism and subsequently to the secondary additive—so called wavelength shifter—which de-excites via emission of photons in the wavelength range shifted towards longer values with respect to the emission spectrum of the primary additive [1].

Plastic scintillators discussed in this article were obtained via bulk polymerization of styrene or vinyltoluene (Sigma Aldrich, pure, about 50 ppm of polymerization inhibitor: 4-tert-buthylcatechol) [24, 25]. There are many ways of producing plastic scintillators from popular, widely available components like PPO, PTP or POPOP [26, 27].

We use 2-(4-styrylphenyl)benzoxazole as a wavelength shifter (WLS) [28]. This chemical compound is obtained in 3-step synthesis followed by the purification process by means of gel chromatography.

**Fig 2:**
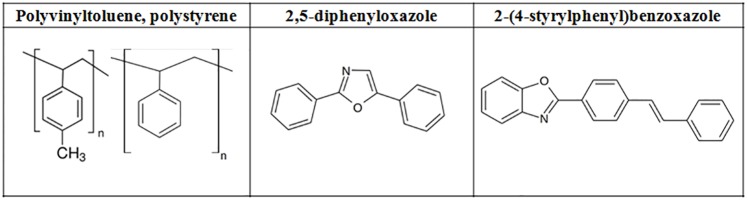
Components of the J-PET plastic scintillators.

Scintillators described in this article, consist of polyvinyltoluene, 2 wt. % of 2,5-diphenyloxazole (Sigma Aldrich, 99%, suitable for scintillation) and various concentrations of 2-(4-styrylphenyl)benzoxazole. Chemical structures of the scintillator components are shown in Fig 2.

The 2-(4-styrylphenyl)benzoxazole is a well known substance which was synthesized for the first time in 1960 by Adolf Emil Siegrist [29]. Since that time it was widely used in many fields e.g. as emitting material in organic light—emitting diodes (OLED) [30] and nonlinear optics (NLO) materials [31], however it was never used as a scintillator dopant so far.

**Fig 3:**
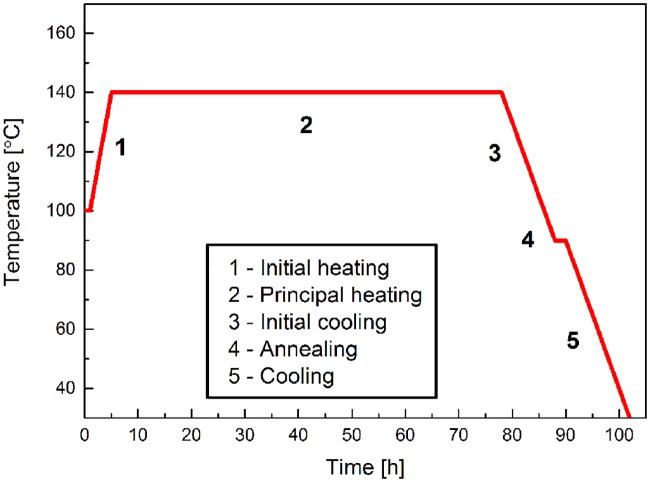
Thermal schedule for scintillator polymerization.

The polymerization process was carried out in silylated glass ampoules. Scintillator dopants were dissolved in liquid purified monomer (styrene or vinyltoluene), next bulk polymerization reaction was initiated thermally. The detailed temperature schedule is shown in Fig 3. The maximal applied temperature reached 140°C and the whole process lasted 100 hours. Obtained scintillators are optically homogeneous. The main disadvantage is polymerization shrinkage covering about 20% of sample volume [32]. It may generate voids or cracks in the block of scintillator. However, when conducting the synthesis with the temperature schedule shown in Fig 3, we obtained pure and homogeneous scintillators characterized by high light output.

### Experimental setup

**Fig 4:**
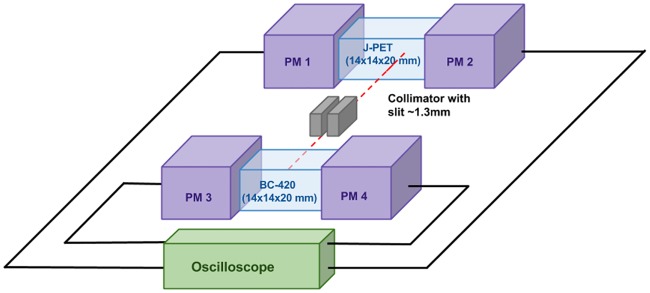
Scheme of the experimental setup for the J-PET scintillators tests. PM denotes photomultiplier. As oscilloscope a Serial Data Analyser (Lecroy SDA6000A) was used. It allowed to collect waveforms of four photomultipliers’ signals simultaneously with the sampling interval of 100 ps.

Measurements of the J-PET scintillators light yield, light signals characteristics and time resolution achievable when applied to the positron emission tomography were carried out with the experimental setup presented in Fig 4. For the tests we used gamma quanta with energy of 511 keV originating from the positron-electron annihilation. ^22^Na radioactive isotope applied as a source of positrons was placed inside a lead collimator providing a well collimated beam of 511 keV gamma quanta with the spatial profile of about 1 mm [33]. The setup enabled to perform simultaneous measurements with the tested and reference scintillator. Coincident registration of signals in reference and tested detector and the 20 cm long slit of the collimator ensured reduction of the background from the 1.27 MeV quanta to the negligible level. For reference we used ternary plastic scintillator BC-420 [22]. The scintillators were cut to the rectangular shape with the dimensions of 14 x 14 x 20 mm^3^. The surfaces of the scintillators were polished. Such prepared samples were wrapped in the Vikuiti reflective foil [34] and placed between Hamamatsu R9800 photomultipliers as shown in Fig 4 [35]. The optical connection between scintillator and photomultiplier was provided by the EJ-550 optical gel [36].

## Results and discussion

In this section basic properties of the J-PET scintillators are presented and compared to properties of the state-of-the art plastic scintillator BC-420 which is used in the first J-PET scanner [22].

### Emission spectra

Photo-induced emission spectrum of a thin sample of J-PET scintillator was measured with spectrophotometer FluoroLog-3 (Horiba Jobin-Yvon). Measurements were carried out in reflecting mode using R928P PMT and continuous wave xenon source.

**Fig 5:**
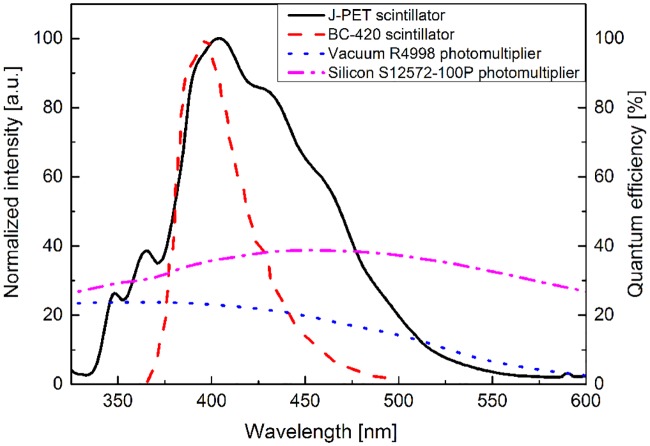
Emission spectra of scintillators and quantum efficieny of photomultipliers. Emission spectra of the J-PET (solid line) and BC-420 (dashed line) scintillators [22] superimposed on the quantum efficiency dependence on photons wavelength for typical vacuum tube photomultiplier with bialcali window (dotted line) [35] and silicon photomultipliers (dashed-dotted line) [35]. Maximum of emission for BC-420 scintillator is placed at wavelength of 393 nm while the maximum of the J-PET scintillator at 403 nm. The emission spectra are normalized in amplitude.

**Fig 6:**
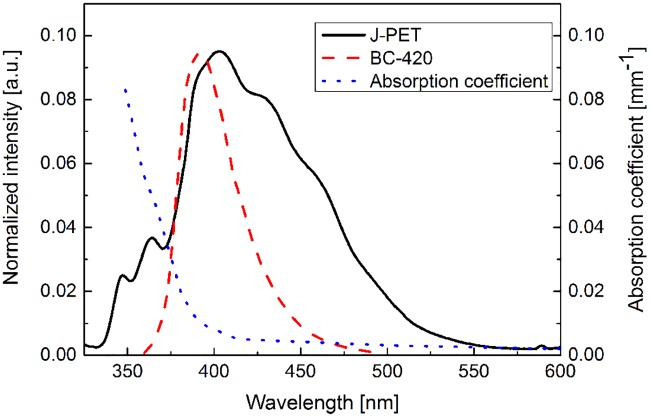
Emission spectra of BC-420, J-PET scintillator and scintillators’ absorption coefficient *μ*_*eff*_ [37, 38]. The emission spectra are normalized in amplitude.

The obtained emission spectrum of the J-PET scintillator is indicated as solid black line in Figs 5 and 6. One can notice that compared to the BC-420 scintillator (red dashed line) the J-PET scintillator emits photons in a significantly broader wavelength range. In particular with respect to the BC-420, the J-PET emission spectrum is extended towards longer wavelengths enabling more effective registration of the scintillation light when using SiPMs whose quantum efficiency is increasing (up to about 450 nm) with the increasing wavelength as indicated by pink dashed-dotted line in Fig 5.

Emission of photons with larger wavelength is beneficial also in view of the light attenuation. In Fig 6 emission spectra for the BC-420 and the J-PET scintillators are compared to the effective light absorption coefficient (blue dotted line) (*μ*_*eff*_) determined for the BC-420 scintillator strips with rectangular cross section of 7 mm x 19 mm [37]. It was established based on the absorption coefficient of pure polystyrene [38] and accounting for losses due to the transport of photons through the scintillator bar [37]. The decrease of the value of absorption coefficient with the growth of the wavelength indicates that the scintillation light for the J-PET scintillator will be less attenuated (with attenuation factor proportional to *exp*(−*μ*_*eff*_) with respect to the BC-420 since the J-PET scintillator spectrum is extended toward higher wavelengths.

### Molecular weight

Temperature schedule applied for the synthesis of scintillator influences not only its homogeneity but also its molecular mass. In our case, pure polystyrene samples were prepared using temperature schedule shown in Fig 3, and their molecular weight measured by means of capillary viscometry is equal to about (2 × 10^5^) u. As it is shown in article [39] light output is changing significantly with the polystyrene molecular weight below about 10^5^ u, and it is constant above this value. Thus large molecular weight of the J-PET scintillator ensures the light output stability and repeatability.

### Light yield

**Fig 7:**
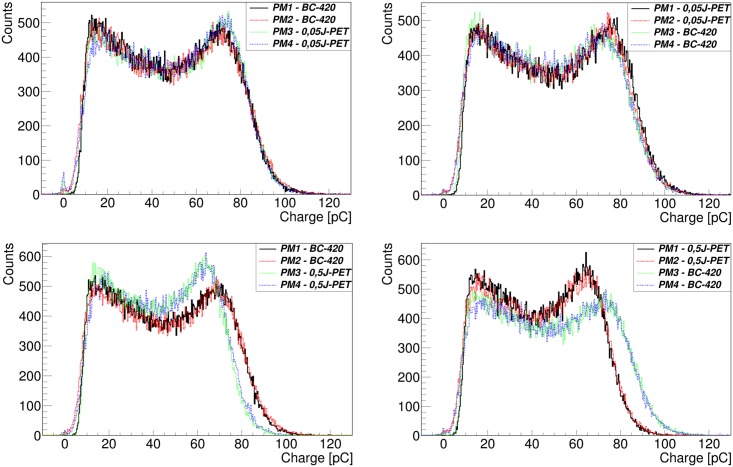
Charge spectra measured with the J-PET and BC-420 scintillator. PM1, PM2, PM3, PM4 used in the legend denote photomultipliers indicated in Fig 3. In the left side charge spectra of the J-PET scintillator (0.05J-PET or 0.5J-PET) measured with photomultipliers PM1 and PM2 are compared to the spectra of BC-420 scintillator measured with the same pair of photomultipliers. The number in the name of the J-PET scintillator indicates the concentration of WLS in ‰. In the right side spectra of the J-PET scintillators are compared to spectra of BC-420 measured with the same pair of photomultipliers: PM3 and PM4. The difference between the spectra for the low values of charge are due to the fact that PM1 and PM3 were used in the trigger. The differences at large charge (at the Compton edge) are due to the differences in the light yield.

Light yield defined as a number of emitted photons per unit of deposited energy is one of the most important characteristics of scintillators. In case of scintillator introduced in this article, it was determined relative to the known light yield of the BC-420 material. For this purpose both: tested and reference scintillators were irradiated simultaneously with the 511 keV gamma quanta. Measurements were conducted by means of the experimental setup described in section Materials and Methods (Fig 4). Exemplary distributions of charge of registered signals are shown in Fig 7. Plastic scintillators are composed mainly of hydrogen and carbon. Due to the low atomic number of these elements, the 511 keV gamma quanta interacts with electrons of the material structure predominantly via Compton effect. Therefore the observed charge distribution is continuous and does not show photoelectric maximum typical in the case of the measurements with crystal scintillators. The charge of the registered signal is proportional to the number of scintillation photons which in turn is proportional to the energy transferred by the interacting gamma quantum to the electrons in the scintillator. For the fixed energy of 511 keV gamma quanta the maximum possible energy transfer is well defined and is equal to 341 keV. Thus by the comparison of the maximum charge of signals from the J-PET and BC-420 we may determine the light yield of J-PET scintillator relative to the light yield of BC-420.

The measurements were conducted for series of eight scintillators each with different mass fraction of the wavelength shifter 2-(4-styrylphenyl)benzoxazole varying from 0 ‰ to 0.5 ‰. The upper and lower panels of Fig 7 show examples of spectra obtained for the mass fractions of WLS equal to 0.05 ‰ and 0.5 ‰, respectively. In order to minimize instrumental uncertainties due to the possible miscalibration of photomultipliers we have performed tests for each sample twice, exchanging the J-PET and BC-420 scintillators in the setup (see Fig 4). In the left panel of Fig 7 the spectra for J-PET and BC-420 scintillators connected to PM1 and PM2 are shown while the right panel shows spectra registered when J-PET and BC-420 scintillator were connected to PM3 and PM4.

Spectra registered by each photomultiplier for BC-420 and the J-PET scintillator were compared to each other. The comparison was carried out using two methods.

The first method, described in details in reference [2], involves calculating the scaling factor by which the charge from BC-420 signals need to be multiplied that the J-PET and BC-420 charge spectra fit together. This scaling factor: *LR* = *Q*_*JPET*_/*Q*_*BC*−420_ is equal to the ratio of light outputs of these scintillators. We have determined the value of LR for each photomultiplier separately, and a mean value of obtained results is shown in the second column of Table 1.

**Table 1:** Relative and absolute light output of the J-PET scintillators as a function of the WLS concentration.

WLS concentration [‰]	LR (Method 1)	LR (Method 2)	Relative light output	Light output [photons/MeV]
0	0.636 ± 0.020	0.625 ± 0.002	0.625 ± 0.002	6397 ± 25
0.025	0.939 ± 0.008	0.938 ± 0.016	0.939 ± 0.007	9613 ± 72
0.05	1.009 ± 0.018	1.006 ± 0.021	1.008 ± 0.014	10318 ± 140
0.1	0.968 ± 0.015	0.967 ± 0.019	0.967 ± 0.019	9902 ± 200
0.2	0.965 ± 0.034	0.962 ± 0.031	0.962 ± 0.031	9853 ± 320
0.3	0.913 ± 0.017	0.912 ± 0.023	0.913 ± 0.014	9346 ± 140
0.4	0.910 ± 0.016	0.910 ± 0.035	0.910 ± 0.015	9317 ± 150
0.5	0.895 ± 0.023	0.897 ± 0.031	0.896 ± 0.018	9172 ± 190

In the second method we calculated *LR* as a ratio of the middles of the Compton edge of the charge spectra for the J-PET and BC-420 scintillators (centre of the right edges of spectra). The middle of the Compton edge was determined by fitting to the edge of the charge spectrum a Novosybirsk function [40]. The result obtained using this method is shown in the third column of Table 1.

Results obtained by both methods are statistically consistent within error bars. In Table 1 a total error including statistical and systematic uncertainties is shown.

Statistical uncertainties of fitting in both methods are of the order of one thousandth of the light output value. They are much smaller in comparison to the systematic uncertainties coming from differences caused by measurements with different photomultipliers. Differences resulting from the use of different fitting methods are also negligible comparing to uncertainties related to measurements with different photomultipliers.

Fourth column of Table 1 includes results obtained as weighted average of light output values determined by Method 1 and Method 2. In the fifth column absolute values of light output are given. They are calculated basing of results from the fourth column and light output of BC-420 scintillator equal to 10240 photons/MeV [22].

**Fig 8:**
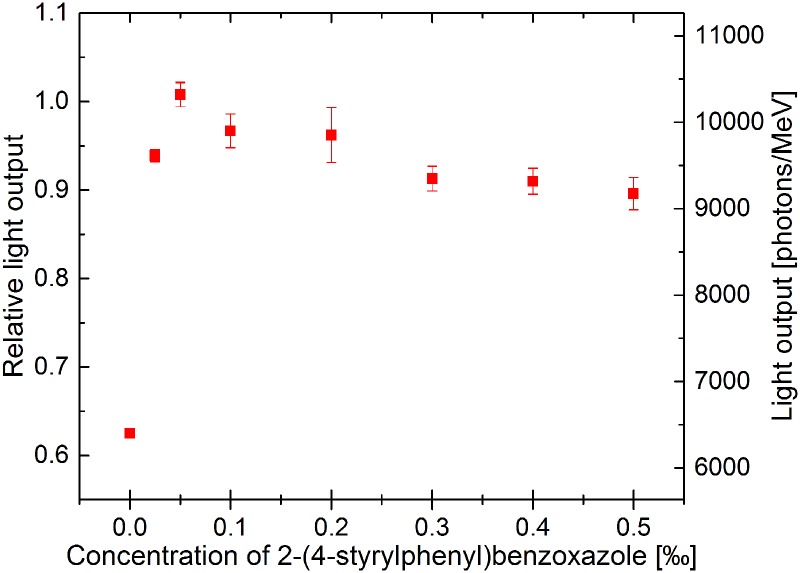
Relative and absolute light output of the J-PET plastic scintillators.

The final result is shown in Fig 8 which indicates that the light output of the J-PET scintillator is the largest for 2-(4-styrylphenyl)benzoxazole concentration of 0.05 ‰. It corresponds to 10318 ± 139 photons per MeV. This result is similar to the light output value of BC-420 scintillator which is equal to 10240 photons/MeV [22]. In case of lower and larger concentrations of WLS, light output gradually decreases. WLS concentrations lower than 0.05 ‰ are insufficient to enable the maximal efficiency. For the higher wavelength shifter concentrations the light yield decreases due to the quenching effect [41].

According to Adadurov et al. [41], optimal concentration of fluorescent additives in plastic scintillators is not a fixed value characterizing the material. Optimization of the scintillator composition has to be done for particular shape and size of the detector.

In the article [41], optimization of commercial wavelength shifter (POPOP) concentration was carried out, however shape and size of tested samples differ from scintillators described in this article, so it is impossible to compare quantitatively concentration and effectiveness of POPOP and our wavelength shifter. Nevertheless, effects observed in both experiments, like unsaturation or concentration quenching are similar.

The minimum light output of about 6400 photons/MeV is observed as expected for the scintillator made solely of polyvinyltoluene and primary fluor PPO.

### Rise and decay times of light signals

**Fig 9:**
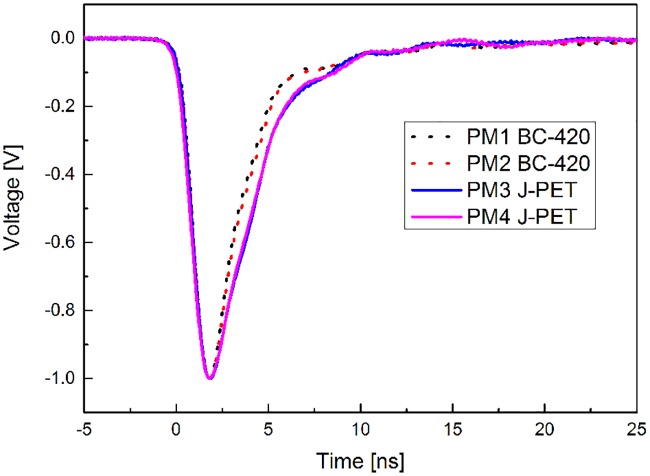
Average signals measured with detectors consisting of the BC-420 or the J-PET scintillators and Hamamatsu R9800 photomultiplier. The shown shapes result from averaging of 10^5^ signals using method described in references [17]. Signals were normalized to the amplitude equal to 1V.

As a main timing characteristics of the J-PET scintillator we determine the rise and decay time of the light signals and compare them to the corresponding characteristics of the BC-420 scintillator. The rise time values were determined calculating time differences between 10% and 90% of the signals amplitude on the leading edge: *t*_10−90_. Fig 9 shows shapes of signals registered by photomultipliers for the 0.05J-PET and BC-420 scintillators. All average signals were normalized to amplitude equal to 1 V.

The shape of signals shown in Fig 9 is a convolution of the temporal density distribution of photons emitted by the scintillator, shape of the single photo-electron signal generated by photomultiplier, transit time spread (TTS) and the broadening of signal due to the finite bandwidth of the oscilloscope. Thus in general the rise time of the light signal (*T*_*scintillator*_) may be extracted from the rise time of the measured electric signal (*T*_*observed*_) using the following formula:

$$\begin{array}{c} T_{scintillator}=\sqrt{T_{observed}^{2}-T_{photomultiplier}^{2}-T_{oscilloscope}^{2}-TTS^{2}-T_{eff}^{2}} \end{array}$$ (1)

where *T*_*photomultiplier*_, *T*_*oscilloscope*_ and *TTS* denote the contribution to the observed rise time due to the shape of the single photo-electron signal, oscilloscope bandwidth and transit time spread, respectively. *T*_*eff*_ denotes the overall effective contributions due to the possible deviation of the nominal values of the above mentioned properties from the values provided by the manufacturers. *T*_*photomultiplier*_ is equal to 1 ns [35], *T*_*oscilloscope*_ was calculated as a ratio 350/bandwidth, and is equal to 0.07 ns while *TTS* value is 0.27 ns [35]. Mean rise time of the electric signals: *t*_10−90_ is equal to 1.22 ± 0.02 ns for BC-420 and 1.24 ± 0.02 ns for the J-PET scintillator, respectively. The rise time of the BC-420 scintillator is equal to 0.5 ns [22]. This implies that within the measurement uncertainties the rise time of the scintillation in the J-PET scintillator is equal to the rise time of the BC-420 scintillator and amounts to 0.50 ns.

Although the signal rise time of BC-420 and the J-PET scintillators are almost equal, as it is visible in Fig 8, there is a slight difference in the decay time in both kinds of scintillators. In order to estimate a decay time of the signal we compare the measured average signals with the theoretical formula accounting for the scintillator emission spectrum and the signal modifications due to the electronic circuit and photomultiplier. The influence of the electronics is clearly visible in Fig 8 as oscillations of the signals at the trailing edge. In ternary plastic scintillators the distribution of the time of the photon emission followed by the interaction of the gamma quantum at time Θ is given by the Formula: described in [42, 43].

$$\begin{array}{c} f\left(t|\Theta \right)=K\int_{\Theta }^{t}\left(exp\left(-\frac{t-\tau }{t_{d}}\right)-exp\left(-\frac{t-\tau }{t_{r}}\right)\right)\cdot exp\left(-\frac{{\left(\tau -\Theta -2.5\sigma \right)}^{2}}{2\sigma^{2}}d\tau \right), \end{array}$$ (2)

where the Gaussian term with the standard deviation *σ* reflects the rate of energy transfer to the primary solute, whereas *t*_*r*_ and *t*_*d*_ denote the average time of the energy transfer to the wavelength shifter, and decay time of the final light emission, respectively. K stands for the normalization constant.

The influence of the electronics on the signal was parametrised effectively by the following formula:

$$\begin{array}{c} N(sin^{2}\left(\alpha t+\varphi \right)+\Delta )exp\left(-\frac{t}{\delta }\right)\cdot f_{Gauss}, \end{array}$$ (3)

where *N*, *α*, *φ*, Δ and *δ* denote fitting parameters and *f*_*Gauss*_ denotes the Gauss function.

**Fig 10:**
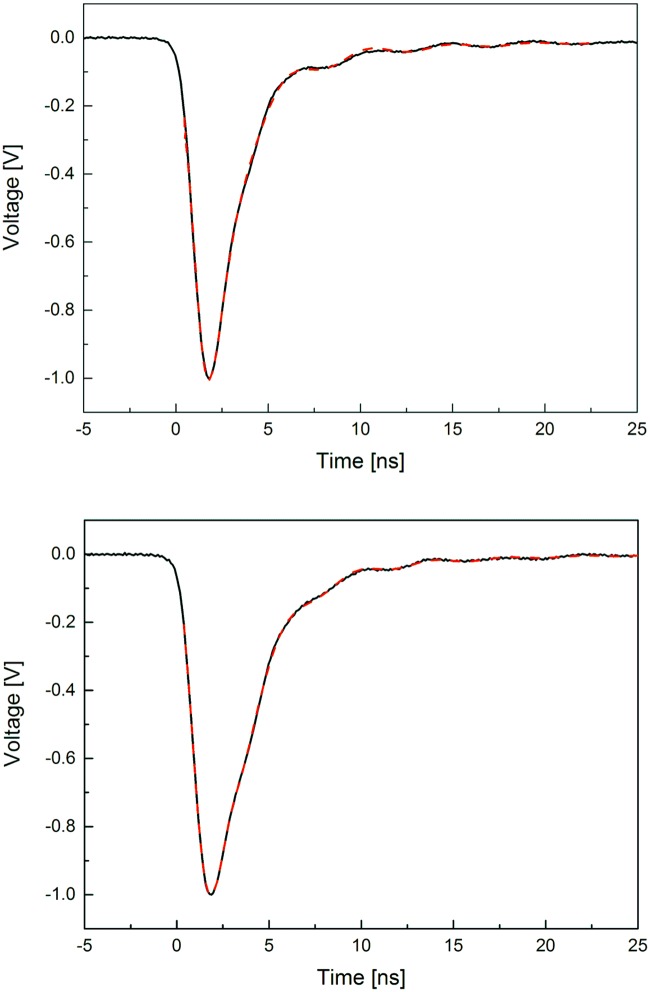
Averaged signals from photomultiplier connected to BC-420 scintillator (top) and to J-PET scintillator (bottom). Red dashed line indicates result of fitting a function given by the sum of Formulas (2) and (3). Signals were normalized to the amplitude equal to 1V.

Averaged signals registered by one photomultiplier connected to BC-420 and J-PET scintillator with the fitted function being a sum of equations given by Formulas (2) and (3) are shown in Fig 10.

Decay time values determined for BC-420 and the J-PET scintillators are equal to 1.49 ± 0.02 ns for BC-420 and 1.91 ± 0.03 ns for the J-PET scintillator, respectively. The value determined for the BC-420 scintillator agrees well with the decay time given by the manufacturer, equal to 1.5 ns [22]. It is worth to mention that the determined decay time of the J-PET scintillator is comparable with the decay time of commercial scintillators, e.g. BC-404, widely used as fast counting detector, with decay time of 1.8 ns and BC-408 with decay time equal to 2.1 ns, respectively.

In Table 2 basic properties of the J-PET and BC scintillators are compared. Light output of J-PET scintillator is equal to light output of commercially available scintillators. Rise and decay time values of the novel scintillator described in this article, are similar to BC scintillators. Maximal wavelength of J-PET scintillator emission and the shape of emission spectra enables the use of the scintillators in novel PET/MRI scanner.

**Table 2:** Properties of J-PET scintillator and of exemplary plastic scintillators manufactured by Saint Gobain [22].

Properties	BC-420	BC-404	BC-408	J-PET
Light output, [% Anthracene]	64	68	64	64
Rise time [ns]	0.5	0.7	0.9	0.5
Decay time [ns]	1.5	1.8	2.1	1.9
Peak emission wavelength [nm]	391	408	425	404
H:C ratio	1.102	1.107	1.104	1.104

## Conclusions

In this article properties of novel J-PET plastic scintillator made of polyvinyltoluene base, doped with 2,5-diphenyloxazole and 2-(4-styrylphenyl)benzoxazole as a wavelength shifter were presented. The purpose of the development was the elaboration of scintillator with optical properties allowing for more efficient transport of photons in long strip geometry, their more efficient registration with SiPM array with respect to the presently available plastic scintillators and, at the same time, with the superior timing characteristics. The novelty of the elaborated scintillator lies in the use of 2-(4-styrylphenyl)benzoxazole as a second additive—wavelength shifter. The substance has been used for the first time as a scintillator dopant. The new scintillator was manufactured via bulk polymerization of vinyltoluene and the optimal concentration of the 2-(4-styrylphenyl)benzoxazole was set by maximizing the light output for particular size and shape of the scintillator. It was shown that the maximum light output of the new scintillator is achieved when concentration of WLS is equal to 0.05 ‰. The light yield for the optimum 2-(4-styrylphenyl)benzoxazole concentration was established to be equal to about 10^4^ photons per MeV. The rise- and decay-times of the developed scintillator were determined to be 0.5 ns and 1.9 ns, respectively. It was shown that when applied to the positron emission tomography it provides time resolution as obtainable with the BC-420 plastic scintillators. Moreover, the emission spectrum of the developed scintillator is extended towards longer wavelengths with respect to the typical commercial plastic scintillators as e.g. BC-420. It is therefore better matched to the quantum efficiency of silicon photomultipliers and lowers the light attenuation. These features of the developed scintillator make it suitable for the application in the construction of the PET insert for the simultaneous PET and MR imaging [18, 19].
